# Risk Factors of Invasive Fungal Infection in Recipients After Liver Transplantation: A Systematic Review and Meta-Analysis

**DOI:** 10.3389/fmed.2021.687028

**Published:** 2021-10-04

**Authors:** Min Liu, Zhijun Zhu, Liying Sun

**Affiliations:** ^1^Department of Liver Transplantation Center, Beijing Friendship Hospital, Capital Medical University, Beijing, China; ^2^National Clinical Research Centre for Digestive Diseases, Beijing Friendship Hospital, Capital Medical University, Beijing, China; ^3^Department of Intensive Care Unit, Beijing Friendship Hospital, Capital Medical University, Beijing, China

**Keywords:** invasive fungal infection, liver transplantation, risk factors, meta-analysis, end-stage liver diseases

## Abstract

**Objectives:** Invasive fungal infection (IFI) remains an important cause of mortality in liver transplantation (LT). The objective of this meta-analysis was to identify the risk factors for IFI after LT.

**Methods:** We searched for relevant studies published up to June 2020 from PubMed, Web of Science, Embase, and the Cochrane Library. Odds ratios (ORs) and their corresponding 95% CIs were used to identify significant differences in the risk factors. Heterogeneity between studies was evaluated by the *I*^2^ test, and potential publication bias was assessed with Egger's test. The quality of included studies was evaluated with the Newcastle-Ottawa Scale (NOS).

**Results:** A total of 14 studies enrolling 4,284 recipients were included in the meta-analysis. Reoperation (OR = 2.18, 95% CI: 1.61–2.94), posttransplantation dialysis (OR = 2.03, 95% CI: 1.52–2.72), bacterial infection (OR = 1.81, 95% CI: 1.33–2.46), live donor (OR = 1.78, 95% CI: 1.20–2.63), retransplantation (OR = 2.45, 95% CI: 1.54–3.89), and fungal colonization (OR = 2.60, 95% CI: 1.99–3.42) were associated with the risk factors of IFI after LT.

**Conclusions:** Despite some risk factors that have been identified as significant factors for IFI post-LT, which may inform prevention recommendations, rigorous and well-designed studies with adequate sample sizes should be conducted to solve the limitations of this study.

## Introduction

Since the first liver transplantation (LT) by Thomas Starzl in 1963, the prognosis was significantly improved. LT can be considered the most effective therapy for nearly all causes of end-stage liver diseases (ESLDs) (1). The incidence of invasive fungal infections (IFIs) in liver transplant recipients is surpassed only by small bowel and lung transplant recipients (2). IFIs occur in 7–42% of patients with liver transplant (3). Despite the advance in surgical techniques and antifungal prophylactic strategies, IFI is still a major cause of postoperative morbidity and mortality for patients undergoing LT (4). The reported mortality associated with IFI range from 25 to 69% (5).

Several clinical trials have shown that the causative organisms of IFIs are variable. *Candida* species are the most common causes of IFI post-LT, followed by *Aspergillus* species (4). Recipients with active or latent infection in the recipient or donor at the time of transplantation, or early graft dysfunction or rejection, are at particularly high risk of developing opportunistic infections (6). Therefore, the incidence of IFI is strongly associated with the hospital microbiological environment, level of immunosuppression, the clinical condition of the patient, surgical factors, complicated transplant operation, and use of high-dose antibiotics (6, 7).

Many studies have reported the risk factors for IFI post-LT, including bacterial infections in the first month and absence of antifungal prophylaxis (7), reoperation, or retransplantation (5). Model for End-Stage Liver Disease (MELD) Score, cytomegalovirus reactivation (8), Roux-en-y anastomosis, and hemodialysis (9). However, because of geographical limitations and sample size, the outcomes of studies of risk factors for IFI post-LT are controversial. For instance, three studies reported that the MELD score was a risk factor for IFI posttransplant (7, 10, 11), but another study reported that the MELD score was not related to IFI post-LT (12). To address the inconsistency in the results, the present meta-analysis measured the quantitative combined effect of all the related studies and increased the power of statistical analysis by merging multiple single studies about the risk factors of IFI in recipients after LT. Finally, this meta-analysis involving 14 studies and provided more reliable evidence for the risk factors of IFI post-LT.

## Methods

### Data Source Collection

A literature search was performed in PubMed, Web of Science, Embase, the Cochrane Library to identify studies related to risk factors of IFI after LT that were published up to June 2020. The terms searched were “liver transplantation or hepatic transplantation or liver grafting” and “invasive fungal infection or IFI” and “risk factors”.

### Inclusion Criteria

Studies were selected in accordance with the “Preferred reporting items for systematic reviews and meta-analyses” (PRISMA) statement (13). The inclusion criteria were as follows: (1) the study was related to the risk factors of IFI in recipients post-LT; (2) IFI was defined as proven or probable according to the European Organization for Research and Treatment of Cancer/Mycoses Study Group (EORTC/MSG) Criteria (14); (3) the study was a case-control or cohort study in design; (4) availability of the published data; (5) the study was written in English.

### Exclusion Criteria

The following were the exclusion criteria: (1) duplicate articles; (2) reviews, meeting abstracts, letters, or case reports; (3) no diagnostic or no defined criteria for IFIs; (4) studies were related to the risk factors of IFIs after the organ transplantation but did not report the relevant data on the LT subgroup; (5) studies included data of risk factors for LT infection but did not show the information for IFI.

### Data Extraction

Relevant information was extracted independently by the two reviewers (ML and LYS). A final check was confirmed by another researcher (ZJZ). The extracted data included the first author, publication year, country of origin, study time and design, number of patients and controls, and risk factors for IFIs with odds ratio (OR) and 95% CI from the multivariate analyses.

### Quality Assessment

The quality of each included study was independently evaluated by the three reviewers (ML, LYS, and ZJZ) based on the Newcastle–Ottawa Scale (NOS) (15). The NOS consists of three domains and a total of nine points: four for selection of study groups, three for outcome and exposure, and two for comparability of the cases. Only studies with scores> 6 were considered as high quality and finally included (16).

### Statistical Analysis

Statistical analysis was performed using Review Manager 5.3 and Stata 12.0. Heterogeneity was considered significant with *I*^2^ > 50% or *P* < 0.1 (17). The fixed-effects model was used to calculate the 95% CI and its pooled ORs for the homogeneous data (*I*^2^ < 50% or *P* > 0.05). Otherwise, the random-effects model was used. Sensitivity analysis was conducted by excluding one study at a time. Publication bias was assessed with Egger's test. *P* < 0.05 suggested that there was a publication bias in this study. The population attributable risk proportion (PARP) was calculated as follows: PARP = *P*_e_ (OR−1)/[*P*_e_ (OR−1) + 1]. *P*_e_ was defined as the pooled exposure rate of controls that represented the overall population exposure rate.

## Results

### Study Selection and Characteristics

**Figure 1:**
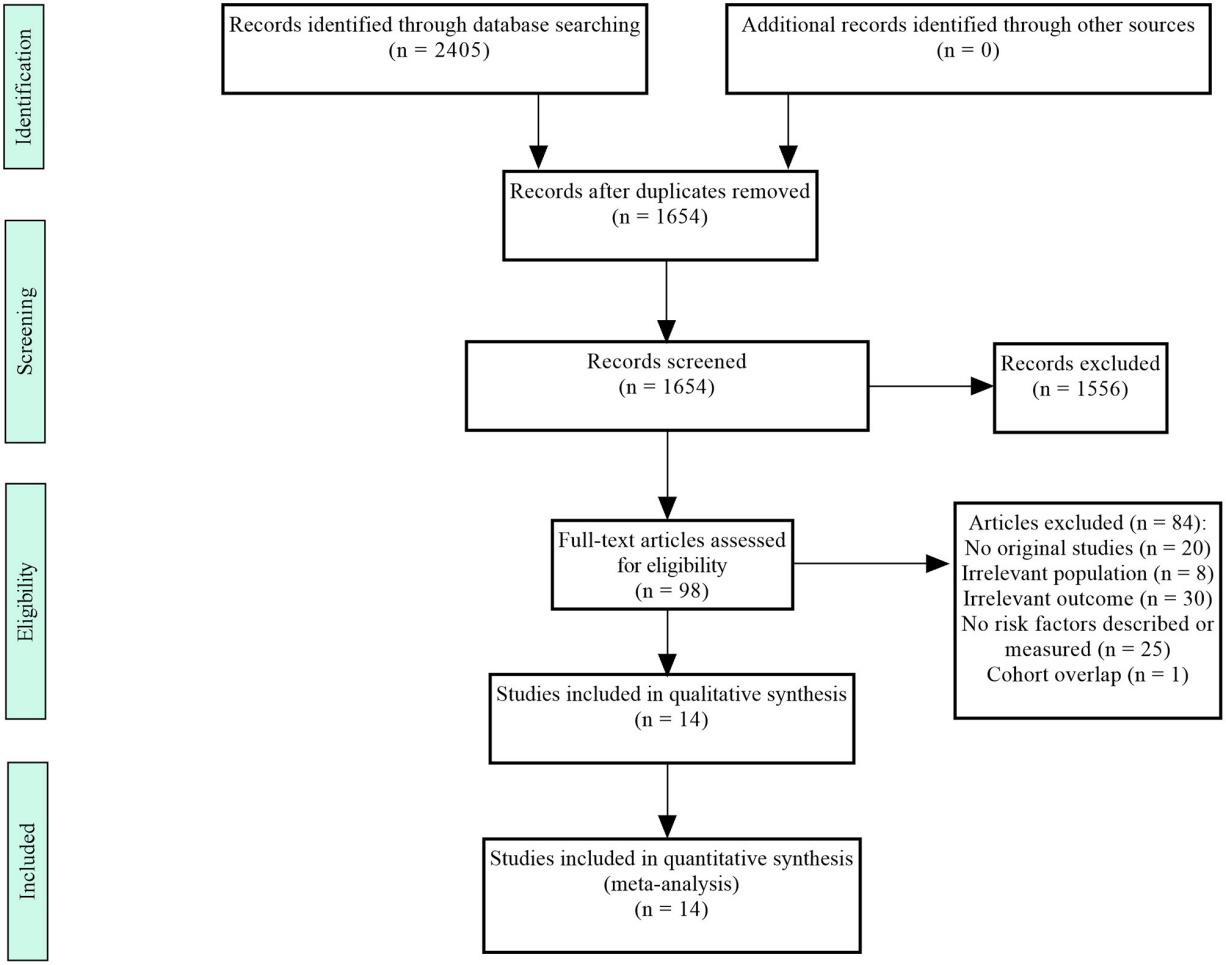
Flowchart of the selection process.

The literature search yielded 2,405 results; most studies were excluded because they were duplicate studies or because they were not relevant to our meta-analysis. Then, 84 studies were excluded after the full-text articles were reviewed because they did not match the criteria. Finally, 14 studies involving 4,284 recipients (533 cases and 3,751 controls) were included in this meta-analysis (7, 10–12, 18–27). The study selection process is shown in Figure 1.

The specific characteristics of the studies included in the meta-analysis are presented in Table 1. These 14 studies were published from 2003 to 2020. They were conducted in the USA (10, 22, 25, 27), Australia (26), China (20), Japan (11, 19, 21), Korea (12), Italy (23, 24), Spain (18), and France (7). A total of seven studies were cohort studies (10, 12, 18, 19, 23, 25, 26) and seven were case–control studies (7, 11, 20–22, 24, 27). All the included studies were evaluated as high quality after being assessed by the NOS.

**Table 1:** Characteristics of studies included in the meta-analysis.

**Study**	**Year**	**Type of study**	**Country**	**Study period**	**N**	**Age (years)**	**Gender (% men)**	**Cases/controls**	**Quality assessment^a^**
Ohkubo et al. (21)	2012	Case-control	Japan	over a 6-year period	156	24.5	46	19/137	6 points
Eschenauer et al. (22)	2015	Case-control	USA	November 2008 to December 2012	382	55.7 ± 10.7	65	20/362	6 points
Kawagishi et al. (19)	2006	Cohort	Japan	July 1991 to November 2005	96	18.67	41	8/88	7 points
Utsumi et al. (11)	2019	Case-control	Japan	January 2005 and April 2012	153	55	54	15/128	8 points
Lavezzo et al. (24)	2017	Case-control	Italy	January 2011 to December 2015	268	NA	NA	16/252	6 points
Fortún et al. (18)	2003	Cohort	Spain	January 1994 to December 2001	131	NA	70	22/109	8 points
Raghuram et al. (27)	2012	Case-control	USA	January 2003 to December2007	502	56	65	58/444	6 points
Lum et al. (26)	2020	Cohort	Australia	January 2005 to October 2015	554	NA	NA	28/56	7 points
Alexander et al. (10)	2006	Cohort	USA	January 1997 to December 1999	153	51	61	28/125	7 points
Kim et al. (12)	2019	Cohort	Korea	January 2009 and February 2012	482	53	76.8	196/286	7 points
Giannella et al. (23)	2016	Cohort	Italy	June 2010 to December 2014	303	53	68.6	19/284	7 points
Jorgenson et al. (25)	2019	Cohort	USA	July 2009 to June 2017	189	54.4 ± 9.9	60.4	50/139	6 points
Zhou et al. (20)	2011	Case-control	China	April 2008 to March 2010	248	50.14 ± 9.68	77.4	44/204	7 points
Saliba et al. (7)	2013	Case-control	France	January 1999 to December2005	667	46.8 ± 13.4	65.6	171/496	7 points

*NA, not available*.

a *High-quality research, 6–9 points*.

### Risk Factors of IFI

**Figure 2:**
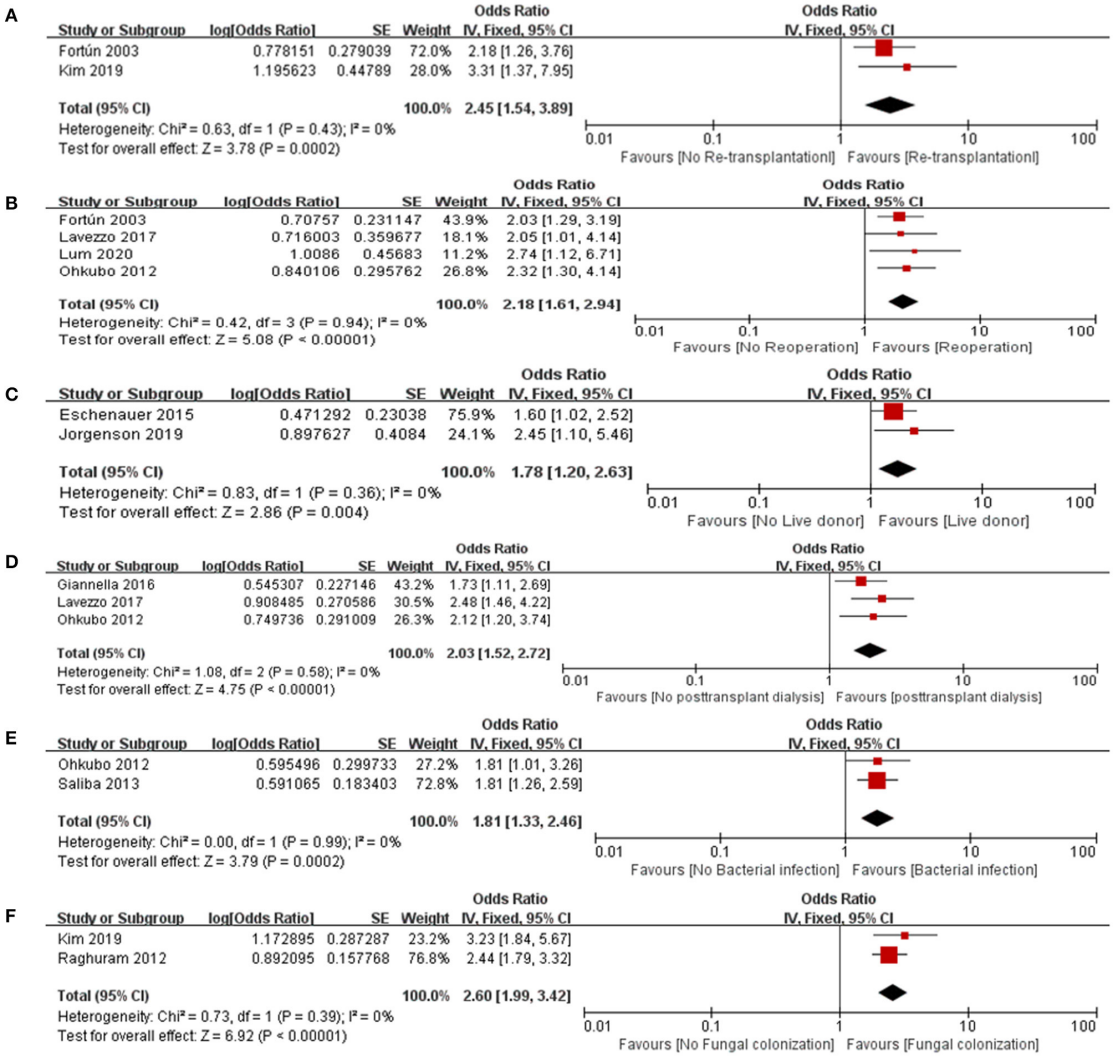
Forest plot for the association between IFI after LT and **(A)** Retransplantation, **(B)** Reoperation, **(C)** Live donor, **(D)** Posttransplant dialysis, **(E)** Bacterial infection and **(F)** Fungal colonization.

The risk factors for IFI post-LT are shown in Table 2. Several risk factors were identified, including reoperation, post-transplant dialysis, bacterial infection, live donor, the MELD score, retransplantation, fungal colonization, Roux-en-Y anastomosis. The risk factors with significant differences in IFIs after LT were as follows: reoperation (OR = 2.18, 95% CI: 1.61–2.94), posttransplantation dialysis (OR = 2.03, 95% CI: 1.52–2.72), bacterial infection (OR = 1.81, 95% CI: 1.33–2.46), live donor (OR = 1.78, 95% CI: 1.20–2.63), retransplantation (OR = 2.45, 95% CI: 1.54–3.89), and fungal colonization (OR = 2.60, 95% CI: 1.99–3.42). A forest plot describing the association between risk factors for IFIs after LT is presented in Figure 2.

**Table 2:** Meta-analysis of risk factors of invasive fungal infection in recipients after liver transplantation.

**Risk factors**	**Combination studies**	**Cases/** **controls**	**OR (95%CI)**	**Z**	***P***	**Heterogeneity of study design**			**Analysis model**	**Egger's test**
						***Chi*** ^**2**^	***p***	***I*** ^**2**^		
Reoperation	4	85/554	2.18[1.61,2.94]	5.08	<0.05^a^	0.42	0.94	0%	Fixed	0.508
Post-transplant dialysis	3	54/673	2.03[1.52,2.72]	4.75	<0.05^a^	1.08	0.58	0%	Fixed	0.747
Bacterial infection	2	190/633	1.81[1.33,2.46]	3.79	0.0002^a^	0.00	0.99	0%	Fixed	NA
Live donor	2	70/501	1.78[1.20,2.63]	2.86	0.004^a^	0.83	0.36	0%	Fixed	NA
MELD score	3	214/749	1.02[0.99,1.05]	1.12	0.26	0.18	0.91	0%	Fixed	0.782
Retransplantation	2	218/395	2.45[1.54,3.89]	3.78	0.0002^a^	0.63	0.43	0%	Fixed	NA
Fungal colonization	2	254/730	2.60[1.99,3.42]	6.92	<0.05^a^	0.73	0.39	0%	Fixed	NA
Roux-en-Y anastomosis	2	199/552	1.83[0.78,4.28]	1.40	0.16	2.64	0.1	62%	Random	NA

a * P < 0.05 stands for significant. NA, not available*.

### PARP of Risk Factors

Population attributable risk proportion was used to represent the proportion of cases in a population that was attributable to the exposed factor. The PARP of risk factors such as reoperation, posttransplant dialysis, bacterial infection, live donor, fungal colonization, and retransplantation is shown in Table 3. The PARP ranged from 6.3 to 37.5%. Reoperation had the highest PARP, whereas live donor had the lowest. Bacterial infection, reoperation, and fungal colonization were strong risk factors for IFIs after LT.

**Table 3:** Population-attributable risk proportion of risk factors of invasive fungal infections in recipients after liver transplantation.

**Risk factors**	**OR (95% CI)**	***P***_***e***_ **(%)**	**PARP (%)**
Reoperation	2.18[1.61,2.94]	36.1	29.9
Posttransplant dialysis	2.03[1.52,2.72]	8.5	8.1
Bacterial infection	1.81[1.33,2.46]	74.1	37.5
Live donor	1.78[1.20,2.63]	8.6	6.3
Retransplantation	2.45[1.54,3.89]	10.6	13.3
Fungal colonization	2.60[1.99,3.42]	14.4	18.7

*OR, odds ratio; CI, confidence interval; P_e_, pool exposure rate; PARP, population-attributable risk proportion*.

### Sensitivity Analysis

Sensitivity analysis was used to evaluate the potential effect of heterogeneity conducted by eliminating one study in each turn. Sensitivity analyses manifested no significant changing of heterogeneity when one study was eliminated at a time.

### Publication Bias

Egger's test was conducted for statistical investigation to evaluate potential publication bias (Table 2). The publication bias was considered to exist for *P* < 0.05. Egger's test showed that most risk factors did not have a publication bias (*P* > 0.05).

## Discussion

Invasive fungal infection is associated with poor outcomes in recipients with posttransplant (28). Therefore, identifying risk factors is essential for preventing IFI post-LT. Accordingly, targeted prophylaxis should be performed only in high-risk recipients (22). Several studies have reported potential risk factors for IFI post-LT. However, there has been inconsistency about the risk factors, perhaps because of the different studies using different designs or inclusion criteria. The current meta-analysis was conducted to identify the risk factors for IFI post-LT and provide the best evidence for the clinical applications.

**Figure 3:**
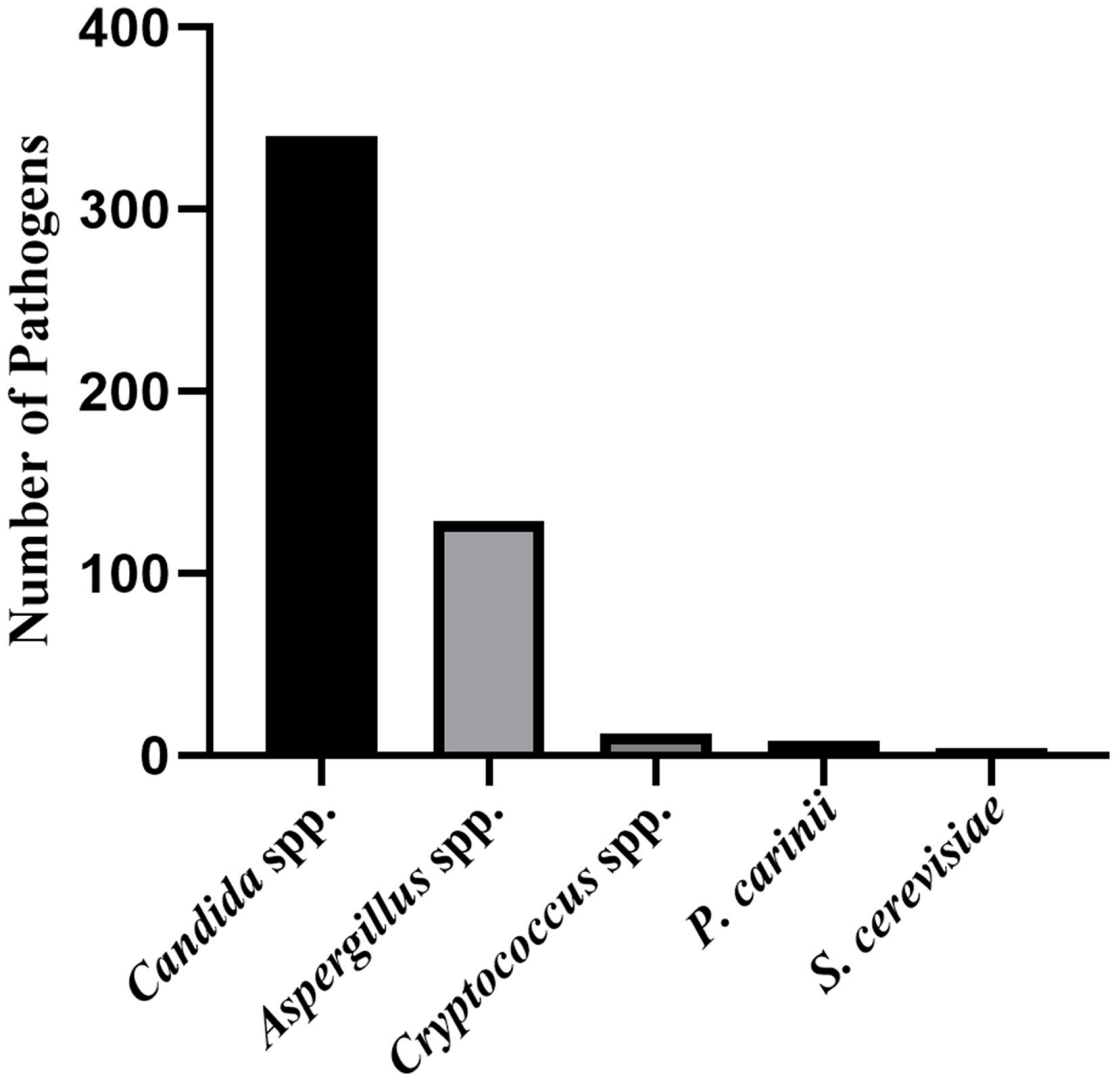
The pathogen composition of IFI after LT. spp, species; *P*. carinit, Pneumocystis cariniee; S. Cerevisiae, Saccharomyces cerevisiae.

According to the inclusion and exclusion criteria, we identified 14 studies enrolling 4,284 patients. In our meta-analysis, risk factors for IFI after LT included reoperation, posttransplant dialysis, bacterial infection, retransplantation, live donor, and fungal colonization. As summarized in Figure 3, 14 studies identified 506 pathogens that caused IFI post-LT. *Candida* species were the most common causative organism of IFI among recipients of the LT, and *Aspergillus* species was the second most common.

Reoperation and retransplantation, which indicated a more complicated intraoperative and postoperative procedure, were risk factors for IFI. Meta-analysis showed that the risk of IFI in recipients with reoperation was 2.18 times higher than the recipients without reoperation, which was consistent with the results of previous studies (29, 30). However, among the studies included in this meta-analysis, Eschenauer et al. (22) and Utsumi et al. (11) concluded that IFI was not associated with reoperation, but the researchers did not give an explanation for this result. Some included studies reported a higher risk of IFI in recipients who underwent retransplantation (12, 18). Our result was consistent with these studies. Several studies reported that retransplantation was not a significant risk factor (22–24), which was likely due to the small number of recipients who underwent retransplantation. The results showed that Roux-en-y anastomosis was not a risk factor for IFI after LT, possibly because of the small number of patients who underwent retransplantation, which was consistent with the results of a previous study (26).

Recipients of the live donor were at a higher risk of IFI after LT, which was consistent with the previous studies (22, 31). In the case of the living donor liver transplant (LDLT), the graft is smaller than that recovered from a deceased donor, and it involves complex surgery and carries a risk of bile leakage. Therefore, antifungal prophylaxis should be given to patients with LDLT to counter the risks of IFI.

Our study also found that the risk of IFI increased with the posttransplant dialysis that is consistent with the studies of Ohkubo et al. (21), Lavezzo et al. (24), and Giannella et al. (23), but several studies (10, 12, 18, 22) found that the posttransplant dialysis was not a risk factor for IFI post-LT; however, the authors did not give any explanation for this negative outcome.

We used PARP to estimate the percentage of IFI in recipients of LT attributed to one kind of risk factor. We found that the PARP of the bacterial infection and reoperation was high. Thus, we infer that these risk factors were important for IFI post-LT.

Fungal colonization was defined as the presence of fungus before LT without clinical symptoms or evidence of infection. We found that the patients with fungal colonization were at a higher risk of IFI. Several studies have shown that antifungal prophylaxis dramatically reduces fungal colonization, mortality caused by a fungal infection, and the overall incidence of fungal infection (5, 32). Further investigation of pretransplant screening to identify fungal colonization is warranted. Therefore, we recommend that post-LT, the recipients should have targeted antifungal prophylaxis to reduce antibiotic exposure.

There were several limitations to this meta-analysis. First, we only included English language literature from four databases, and there may have been incomplete retrieval. Second, there may have been mistakes in the data conversion because some study data required to be recalculated. Third, because of the limitations of the included date, we did not conduct subgroup analyses and funnel plots. At last, analyses were limited by the sample size included in this meta-analysis, so the combined effect size may have been exaggerated to draw the opposite conclusion.

In conclusion, this meta-analysis identified some risk factors for IFI post-LT and might provide a basis for clinical prevention. However, a well-designed prospective cohort study should be conducted to validate our findings.

## Data Availability Statement

The original contributions presented in the study are included in the article/supplementary material, further inquiries can be directed to the corresponding author/s.

## Author Contributions

ML and LYS designed the study and wrote the first draft of the manuscript. ML and ZJZ verified data extraction, data analysis, and reviewed the manuscript. LYS and ZJZ supervised the data acquisition, data analysis, and interpretation. All the authors read and approved the final manuscript.

## Funding

This research was supported by the Beijing Municipal Science & Technology Commission (No. Z181100001718220).

## Conflict of Interest

The authors declare that the research was conducted in the absence of any commercial or financial relationships that could be construed as a potential conflict of interest.

## Publisher's Note

All claims expressed in this article are solely those of the authors and do not necessarily represent those of their affiliated organizations, or those of the publisher, the editors and the reviewers. Any product that may be evaluated in this article, or claim that may be made by its manufacturer, is not guaranteed or endorsed by the publisher.
